# Saturated Fat Intake Is Associated with Lung Function in Individuals with Airflow Obstruction: Results from NHANES 2007–2012

**DOI:** 10.3390/nu11020317

**Published:** 2019-02-01

**Authors:** Kasey Cornell, Morshed Alam, Elizabeth Lyden, Lisa Wood, Tricia D. LeVan, Tara M. Nordgren, Kristina Bailey, Corrine Hanson

**Affiliations:** 1 Internal Medicine Pulmonary, University of Nebraska Medical Center, Omaha, NE 68198, USA; kasey.cornell@unmc.edu (K.C.); tlevan@unmc.edu (T.D.L.); kbailey@unmc.edu (K.B.); 2 College of Public Health, University of Nebraska Medical Center, Omaha, NE 68198, USA; morshed.alam@unmc.edu (M.A.); elyden@unmc.edu (E.L.); 3 School of Biomedical Sciences and Pharmacy, University of Newcastle, Newcastle NSW 2308, Australia; lisa.wood@newcastle.edu.au; 4 College of Public Health Epidemiology, University of Nebraska, Omaha, NE 68198, USA; 5 Research Service, VA Nebraska-Western Iowa Health Care System, Omaha, NE 68105, USA; 6 Division of Biomedical Sciences, University of California Riverside, Riverside, CA 92521, USA; tara.nordgren@medsch.ucr.edu; 7 Medical Nutrition Education, College of Allied Health Professions, University of Nebraska Medical Center, Omaha, NE 68198, USA

**Keywords:** COPD, saturated fat, lipids, lung function, NHANES

## Abstract

Nutritional status is a well-recognized prognostic indicator in chronic obstructive pulmonary disease (COPD); however, very little is known about the relationship between lung function and saturated fat intake. We used data from the cross-sectional National Health and Nutrition Examination Surveys (NHANES) to assess the relationship between saturated fatty acid (SFA) intake and lung function in the general US adult population. Adults in NHANES (2007–2012) with pre-bronchodilator spirometry measurements and dietary SFA intake were included. Primary outcomes were lung function including forced expiratory volume in one second (FEV_1_), FEV_1_, forced vital capacity (FVC), FEV_1_/FVC ratio, percent predicted FEV_1_ and percent predicted FVC. Multivariable regression models in the general population as well as those with spirometry-defined airflow obstruction were used to assess the relationship between lung function measurements and dietary SFA intake after adjustment for confounders. 11,180 eligible participants were included in this study. Univariate analysis revealed a statistically significant positive association between total SFA intake and lung function outcomes; however, these relationships were attenuated after adjustment for covariates. A secondary analysis of individuals with spirometry-defined airflow obstruction (FEV_1_/FVC < 0.7) revealed that a lower intake of SFA was associated with reduced FEV_1_ (β = −126.4, *p* = 0.04 for quartile 1 vs. quartile 4), FVC (β = −165.8. *p* = 0.01 for quartile 1 vs. quartile 4), and percent predicted FVC (β = −3.3. *p* = 0.04 for quartile 1 vs. quartile 4), after adjustment for relevant confounders. No associations were observed for the FEV_1_/FVC ratio and percent predicted FEV_1_. It is possible that characteristics such as food source and fatty acid chain length may influence associations between saturated fatty acid intake and health outcomes.

## 1. Introduction

The two major diagnoses associated with obstructive lung disease in the United States (US) are asthma and chronic obstructive pulmonary disease (COPD). Of these, COPD is a leading cause of death in the US and has been on the rise since 1980 [1]. In addition to smoking cessation, there have been efforts to reduce the number of deaths due to respiratory disease using other modifiable risk factors, including diet. Several different dietary factors have been evaluated with regard to lung function and respiratory diseases, including individual micronutrients [2,3], fiber [4], polyunsaturated omega-3 and -6 fatty acids and dietary patterns [5,6]. 

One group of nutritional compounds, the polyunsaturated fatty acids (PUFA) omega (*n*)-3 and *n*-6 fatty acids, have received much attention regarding their potential role in respiratory disease. These fatty acids are precursors to potent lipid mediator signaling eicosanoid molecules, which have important roles in the regulation of inflammation. Eicosanoids derived from *n*-6 PUFA are generally considered pro-inflammatory while eicosanoids derived from *n*-3 PUFA are anti-inflammatory [7]. Several studies support the notion that diets high in *n*-3 fatty acids may be beneficial in inflammatory lung conditions, including asthma [8,9] and COPD [10,11]. Some studies have shown that increased *n*-3 intake is inversely related to the risk of COPD in a quantity-dependent fashion [11]. Additionally, lung function is improved in people with increased fish intake, suggesting that *n*-3 rich foods, such as fish, have a positive effect on lung function [12]. Other studies have not shown a protective effect of *n*-3 in relation to COPD or asthma [13], however a randomized controlled trial involving COPD patients showed improved exercise tolerance and reduced serum inflammatory markers compared to controls after supplementation with *n*-3 PUFA for eight weeks [14]. 

Saturated fatty acids (SFA) have received much less attention in relation to lung disease. Overall, increased intake of dietary fat as a percentage of calories has been associated with impaired lung function in older men, with this affect attributed to chronic over-activation of the innate immune system [15]. High levels of SFA intake have been strongly associated with the development of cardiovascular disease. This may be, in part, because high amounts of SFA intake negatively affect the blood lipid profile by increasing the level of low-density lipoprotein, which is an acknowledged biomarker of risk for cardiovascular disease [16]. Current evidence also suggests that SFAs act as non-microbial TLR4 agonists and trigger an inflammatory response via activation of the NF-κB pathway [17]. SFA can also trigger a pro-inflammatory response via upregulating NLRP3 inflammasome activity [18]. Both pulmonary and systemic chronic inflammation is considered a hallmark characteristic of COPD and has been shown to cause a decline in forced expiratory volume in one second (FEV_1_) [19,20]. Mouse models have shown that saturated fat intake increases lung alveolar macrophages and augments airway inflammation [21]. In patients with asthma, consumption of a single high-fat meal leads to increased circulating fatty acid levels, and this corresponds with an increase in neutrophils and TLR4 mRNA expression in cells collected from induced sputum [22]. However, recent evidence has shown that the relationship between SFA intake and disease may be more complex than originally thought, with variations in disease risk based on SFA carbon chain length and the source of the fatty acids [23,24,25].

The National Health and Nutrition Examination Surveys (NHANES) include spirometry measures and dietary intake information in a representative sample of US adults, allowing evaluation of the relationship between saturated fat intake and lung function parameters [26]. Therefore, the purpose of this study was to examine if dietary intake of saturated fat is associated with measures of lung function in a generalizable adult population, as well as a cohort of COPD patients. 

## 2. Methods

### 2.1. Subjects 

This analysis included adults aged 19 years and older from the NHANES (2007–2012) who had data on saturated fat intake as well as pre-bronchodilator spirometry measurements. Pregnant individuals were excluded as well as subjects who had energy intakes outside of a plausible range (<600 or >6000 kcal/day for women; <800 or >8000 kcal/day for men). Detailed methods and protocols for the NHANES study are described within the website reference, including informed consent procedures for all participants [26]. As the data used in our study are freely available in the public domain, the study was exempt from human subject review.

### 2.2. Lung Function Outcomes 

Pre-bronchodilator spirometry was performed from 2007 to 2012. Lung function outcomes included: forced expiratory volume in one second (FEV_1_), forced vital capacity (FVC), FEV_1_/FVC ratio, FEV_1_ percent predicted, and FVC percent predicted. Protocols for these measurements are summarized within the NHANES resources [26]. Lung function was expressed as a percent predicted based on age, gender, height and race (white, black or Mexican/Hispanic) from the third NHANES reference values [27]. There was a sub-group of individuals with post-bronchodilator spirometry; however, few participants had this maneuver performed (955/11,180; 8.5%) so this analysis was based on pre-bronchodilator values.

### 2.3. Respiratory Phenotype Determination 

Criteria for obstructive lung disease was determined via Global Initiative for Chronic Obstructive Lung Disease (GOLD) to assess the presence of airflow obstruction [28]. Our analysis therefore included evaluation of the relationship between SFA intake and lung function measures in two populations: the entire NHANES cohort; and those with airflow obstruction (FEV_1_/FVC < 0.70) where severity was defined according to FEV_1_% predicted into GOLD stages 1-4.

### 2.4. Dietary Assessment 

Dietary intakes in the NHANES survey were determined from two interviewer-administered 24-h recalls, developed and validated by the U.S. Department of Agriculture. Our primary intake of interest was saturated fat. Each participant’s total SFA intake was calculated as grams per day (gm/day) as an average of the 2 days of dietary recall in those that had 2 days available. If the individual had only 1 day of dietary recall available, then the results from 1 day were used. Intakes of individual SFAs were also available within the NHANES data set and included butanoic acid (C4:0), hexanoic acid (C6:0), octanoic acid (C8:0), decanoic acid (C10:0), dodecanoic acid (C12:0), tetradecanoic acid (C14:0), hexadecenoic acid (C16:0), and octadecanoic acid (C18:0). 

### 2.5. Other Covariates 

Potential confounders were identified *a priori* and included age, gender, race, height, energy intake, poverty-to-income ratio (PIR), body mass index (BMI), C-reactive protein (CRP) as a biomarker of inflammation, and smoking status. Smoking status was characterized as never, current (smoked >100 cigarettes in a lifetime and currently smoking), or former (smoked >100 cigarettes in lifetime, but does not currently smoke). BMI categories were created based on the World Health Organization (WHO) BMI classifications and participants were categorized as follows: underweight: <18.5, normal range: 18.5–24.9, overweight: 25–30, and obese: >30. To further examine any confounding effects of extreme obesity, BMI measurements were also categorized as <30 kg/m^2^, >30 kg/m^2^, <40 kg/m^2^ and >40 kg/m^2^.

### 2.6. Statistical Analysis 

Means and standard error were used for descriptive statistics. To incorporate the complex, multistage sampling design of the NHANES in the statistical analysis, the SAS procedures SURVEYFREQ, SURVEYMEANS, SURVEYREG, and SURVEYLOGISTIC were used. Univariate and multivariate regression models were used to evaluate the association of lung function measurements with total SFA intake, quartiles of SFA intake, as well as individual types of SFAs in the entire cohort. SFA intake in quartiles was considered in the models both as a continuous variable and as a categorical variable with quartile 4, highest intake, as the reference group. The multivariable regression models for FEV_1_, FVC, and FEV _1_/FVC ratio were adjusted for possible confounders including age, height, energy intake, poverty to income ratio (PIR), and CRP as continuous variables and gender, race, BMI and smoking status as categorical variables. Models for the outcomes of % predicted FEV_1_ and FVC were adjusted for the confounding factors of energy intake, PIR, BMI, CRP, and smoking status.

As a secondary analysis to examine the relationship between saturated fat intake and lung function in individuals with airway obstruction, we defined participants as: airflow obstructed (FEV_1_/FVC ratio of <0.7) and non-obstructed (FEV_1_/FVC ratio of ≥0.7). As our population included adults ages 19–40 who are more likely to have asthma, we also performed a sensitivity analysis of those over 40 years of age. 

To further examine the effects of obesity on these relationships, we also classified obesity status as BMI ≤30 vs. >30 kg/m^2^; and ≤40 vs. >40 kg/m^2^. We compared lung function outcomes within each stratified group using multivariable regression models adjusted for relevant confounders as described above. 

As smoking remains the most important cause of respiratory disease, we analyzed the interaction between quartiles of saturated fat intake and smoking status. The interaction terms were calculated using SFA quartiles and smoking as a categorical variable. In addition, it is possible that diet may affect lung function differently based on gender [4], therefore a test of interaction between saturated fat intake and gender was conducted. Lastly, an interaction of SFA quartiles and BMI classification also was considered. If any of these interactions were statistically significant, a stratified analysis was conducted to further explore the effect of the interaction on lung function. A p-value of ˂0.05 was considered statistically significant. 

## 3. Results

The final number of eligible participants included in the analysis was 11,180. Overall, 49.5% of the eligible participants were men, with an overall mean age of 44.4 years and a mean BMI of 28.7 kg/m^2^. Never smokers represented 54.5% of the population, whereas 22.9% were former smokers and 22.9% were current smokers. The mean SFA intake of the cohort was 27.6 grams/day, representing 11% of total calories. The demographic characteristics of the sample by quartile of saturated fat intake are represented in Table 1. Compared to those in the highest quartile of saturated fat intake, those in the lowest quartile of saturated fat intake were older, female, and had a lower poverty-to-income ratio. 

In the univariate analysis of the entire cohort, there was a significant positive association noted between total saturated fat intake and FEV_1_ and FVC (β = 17.9 ml and 23.6 ml, *p* ˂ 0.0001 for both), however after adjustment for confounders, these relationships were no longer significant (data not shown). There were statistically significant relationships between lung function measurements and SFA intake in the sub-population of participants who met the spirometry criteria for a diagnosis of airway obstruction (*n* = 1,424). After adjustment for relevant confounders, those in the lowest quartile intake of SFA demonstrated lower FEV_1_, FVC, and percent predicted FVC when compared to the highest quartile of intake (−126.4 ml, −165.8 ml, and −3.3 ml for FEV_1_, FVC, and % pred FVC, respectively, *p* = 0.04, 0.01 and 0.04). No relationship was observed between SFA intake and FEV_1_/FVC ratio or percent predicted FEV_1_. These results are shown in Table 2. In the sensitivity analysis including just those participants over 40 years of age (*n* = 1,221), the overall adjusted results showed that effect sizes remained basically unchanged for FEV_1_ (β = −125.9, *p* = 0.06 for quartile 1 vs. quartile 4), FVC (β = −189.8, *p* = 0.02 for quartile 1 vs. quartile 4), and percent predicted FVC (β = −4.1, *p* = 0.05 for quartile 1 vs. quartile 4). Results for FEV_1_/FVC ratio and percent predicted FEV_1_ also remained similar (β = −0.005, *p* = 0.58, and β = −3.4, *p* = 0.23 for FEV_1_/FVC ratio and percent predicted FEV_1_, respectively). 

When an analysis for interaction between quartiles of saturated fat intake and BMI was conducted, a significant effect for percent predicted FVC was found (*p* for interaction = 0.03) after adjustment for confounders. Specifically, for participants in the BMI range of 25 to <30, FVC percent differed between quartile 2 and quartile 4 of saturated fat intake (β = 1.93, *p* = 0.027) and between quartile 3 and quartile 4 of saturated fat intake (β = 2.34, *p* = 0.0088) after adjusting for the other covariates in the model. There were no significant interactions between gender or smoking and saturated fat intake and lung function outcomes. (Data not shown). 

We next evaluated intakes of individual fatty acids to determine if specific individual fatty acids (C4:0 to C18:0) were driving the observed association between SFA intake and lung function parameters in individuals with airflow obstruction. Intakes of individual saturated fatty acid quartile ranges are shown in Table 3a. In this analysis, we identified a reduction in % predicted FVC associated with intakes in the lower quartiles of butanoic (C4:0), hexanoic (C6:0), octanoic acid (C8:0), decanoic (C10:0), dodecanoic (C12:0) and tetradecanoic (C14:0) acid when compared to the highest quartile of intake (quartile 4, reference group), after adjustment for relevant confounders. Tests for linear trends across these quartiles were also significant (Table 3b). There were no significant associations between the intakes of individual fatty acids and FEV_1_, FVC, FEV_1_/FVC ratio or % predicted FEV_1_ (data not shown). 

## 4. Discussion

To our knowledge, this is the first study to examine associations between lung function and intake of total and individual SFA in adults with and without airway obstruction. In this cross-sectional, United States population-based NHANES sample, we found trends for associations with percent predicted FEV_1_ and total SFA intake in the general population after adjustment for confounding variables. Furthermore, in people with spirometry-defined airflow obstruction, a higher intake of SFA was associated with significantly higher measures of FEV_1_, FVC, and percent predicted FVC. Our results build on similar beneficial associations seen in the literature between SFA intake and other disease states, including an analysis of the European Prospective Investigation into Cancer and Nutrition-Netherlands cohort (EPIC-NL), which showed that higher SFA intake did not increase the risk of ischemic heart disease; indeed, a higher intake of energy from SFA was associated with a 17% lower risk of ischemic heart disease [23].

One plausible explanation for our findings can be found in earlier studies that demonstrate benefits of increased overall intakes of fat in patients with COPD. The suspected etiology of these findings rests in the increased demand for macronutrient substrates by COPD patients, which exists because of their higher basal metabolic rate due to increased work of breathing [29]. As the demand for energy is increased, a large amount of oxygen is consumed and carbon dioxide (CO_2_) is produced from the metabolism of carbohydrates; an effect which is alleviated when fatty acids are used as the energy substrate [30]. As patients with COPD demonstrate impaired ability to exhale CO_2_, metabolism of energy from fatty acids and reduction in CO_2_ can reduce negative consequences such as lethargy and dyspnea [31]. Therefore, it is possible that a higher saturated fat intake was a surrogate for higher overall fat intake, and influenced our findings. 

Supporting this hypothesis, one study revealed reduced CO_2_ production after individuals consumed a high fat, low carbohydrate oral supplement as part of their diet for three weeks, with a resulting improvement in FEV_1_, which is usually associated with obstruction severity [32]. Still another study demonstrated an improved FVC (which is usually associated with restriction) in a group of COPD patients with high-fat, low-carbohydrate supplementation during an acute exacerbation [33]. From these results it is not possible to determine if the findings relate to improvements in airflow restriction or an improvement in respiratory and accessory muscle strength, endurance, and overall effort due to the improvement in their nutrition status [34]. Nonetheless, the data suggest an explanation for the differences seen in our study between participants with COPD and the general population, who eliminate excess CO2 easily [35].

It is interesting to speculate that the chain length of saturated fatty acids may have a more profound impact on disease risk than has been previously thought. The short-chain fatty acids (SCFA), including acetate (C2:0), propionate (C3:0) and butyrate (C4:0), were shown to exhibit anti-inflammatory effects, mediated by immune cell migration and adhesion and suppression of pro-inflammatory signaling pathways such as nuclear factor-κB via inhibition of histone deacetylases and activation of free fatty acid receptors (GPR41 and GPR43) [36,37]. Systemic inflammation is considered an important sub-phenotype of COPD [38,39], and circulating inflammatory biomarker levels were shown to have an inverse relationship with lung function and respiratory morbidity [38]. Therefore, nutrients with potential anti-inflammatory activity could theoretically benefit individuals with COPD. A decreased risk of ischemic heart disease with increased intake of SFA in the EPIC-NL study appeared to be driven primarily by intake of short and medium chain SFAs [23]. Another analysis of NHANES data showed that while long chain SFAs (C14:0–C18:0) were positively associated with an increase in BMI, medium chain SFAs (C12:0) were not [40]. A study of 73,147 women and 42,635 men from the Nurses’ Health Study and the Health Professionals Follow-up Study showed that while higher total SFA intake was associated with an increased incidence of coronary heart disease, this risk was primarily attributed to the longer-chain (C:16) SFA [41]. Similar findings were reported in another analysis of the Nurses’ Health Study, which found that intake of major medium-long chain SFAs (including 12:0, 14:0, 16:0, and 18:0) were associated with an elevated risk of coronary heart disease, whereas the sum of shorter chain fatty acids, including butyric acid (4:0), caproic acid (6:0), caprylic acid (8:0), and capric acid (10:0) was not [42]. When assessing the associations of individual SFA on lung function outcomes in this study, we identified statistically significant reductions in percent predicted FVC in individuals with specific SFA intakes in the lower quartiles of intake compared to the fourth (highest) quartile of intake for several SFAs. The individual fatty acids with significant associations with lung function included all the SFAs with a chain length less than 14 carbons, which indicates that shorter chain fatty acids may provide a protective effect in the lungs. Interestingly, several of the SFAs that were identified as protective in our study are only consumed in small quantities. For example, in our cohort, butanoic acid (C4:0) contributed only 2.5% of the total saturated fatty acid intake. This suggests that these shorter chain fatty acids may be biologically active and can have important effects, even when consumed in small doses. Further work is needed to better understand the required intake of short-medium SFAs to provide a protective, anti-inflammatory effect in the lungs.

It is also important to note that one of the primary sources of SCFAs in humans is the gut microbiome [36]. These microbiome-produced SCFAs have demonstrated anti-inflammatory activities [43]. Increased serum concentrations of SCFAs have been shown to protect against airway allergic inflammation in mouse models [43]. Mouse models have also indicated that the fat content of the diet could alter the gut microbiome, with one study showing that a high-fat diet resulted in a relative increase in bacteria from the phyla Firmicutes, which are SCFA producers [44,45]. It could be hypothesized that butyrate production by the gut microbiome would be enhanced in individuals with increased saturated fat intake, as they experience a change in gut microbial species which increases their ability to produce SCFA [46,47,48,49].

The food source of SFA also appears to be important when assessing relationships to health outcomes. Findings from the EPIC-NL study demonstrated a decreased risk of ischemic heart disease with increased intake of dairy products (milk, cheese, and butter) as the source of SFA [23]. Other studies have also shown a beneficial effect of dairy products on COPD, including an evaluation of the Multi-Ethnic Study of Atherosclerosis (MESA) cohort which showed a decline in CT-defined emphysema with increased intake of dairy products, although the effects were more pronounced with low-fat dairy products [50]. A reduction in cardiovascular disease risk has also been noted when meat in the diet is replaced by dairy products as the source of SFA [25]. A recent randomized controlled trial showed that SFAs from cheese and butter had no effect on several non-lipid cardiometabolic risk factors compared with the effects of carbohydrates and unsaturated fatty acids [24]. The positive effects of dairy products may again relate to fatty acid chain length, as dairy foods contain a higher proportion of medium chain SFA (C:6-C:12), compared to other animal sources such as meat. Indeed results from the current study demonstrate a protective effect of C10:0 and C12:0 on lung function. However, it is also likely that other non-fat nutrients obtained from dairy products, such as minerals and protein, contribute a beneficial effect. Hence further work is needed to understand the impact of whole foods and nutrient combinations on lung health.

Our study has several limitations. Self-reported dietary intake data collected by 24-h recall is subject to recall error and a systemic bias of subjects who avoid reporting their actual intake. It is, however, considered to be the least biased method for describing dietary intake at the population level [51]. It is also likely there are sources of bias and residual confounding not addressed by our methodology. It is also possible that inflammatory biomarkers other than CRP mediate these relationships. The NHANES data we analyzed is cross-sectional, so we were not able to evaluate any temporal relationships, such as dietary effects on lung function decline, nor can we establish causality. Because intake of fat helps in the digestion and absorption of fat-soluble vitamins including vitamins D, E, and A that have been associated with improvement in lung function, any protective effect of higher fat intake in our cohort may be attributable to increased absorption of these nutrients. Types of foods may also be important in considering outcomes. Saturated fat is found mainly in animal products such as red meat and dairy; however, the distribution of short and long chain SFA differs significantly between these food groups [52]. Intakes of different food groups was not evaluated as part of our study. Adjustment for physical activity, which is relevant to dietary choices, was also not adjusted for in this analysis and remains a potential source of confounding. This study used pre-bronchodilator measurements for lung function rather than post-bronchodilator values, so it is more difficult to extrapolate our findings on saturated fat intake and lung function in this study to COPD. It is also possible that our use of a fixed ratio to define COPD may have caused misclassification of some participants; however, we chose the GOLD criteria over a lower limit of normal (LLN) classification to avoid false negatives in an elderly population, who are more likely to have COPD [53]. Additionally, GOLD and LLN criteria have been shown to demonstrate associations with important health outcomes to a similar magnitude [54]. Our study does have a major strength in our ability to use spirometry measurements for identifying the presence of airflow obstruction, as opposed to the self-reported diagnosis of COPD used in many epidemiological studies. 

## 5. Conclusions

We have observed an increased in lung function with increased SFA intake in individuals with COPD. The precise mechanisms driving this association are not known, however our findings highlight the possibility that different types of SFA have different effects on disease risk. More research into the impact of SCFA of different chain length, from food sources, will be important in developing dietary recommendations around fat intake for COPD.

## Figures and Tables

**Table 1:** Participant characteristics stratified by the total saturated fat intake quartiles (*n* = 11,180).

Characteristic	Entire population Mean (SE)	Quartile 1 Mean (SE) ≤16.98 g/day	Quartile 2 Mean (SE) >16.98 to 24.06 g/day	Quartile 3 Mean (SE) >24.06 to 33.69 g/day	Quartile 4 Mean (SE) >33.69	*p* Value
**Continuous Variables**						
Age (years)	44.4 (0.40)	46.1 (0.44)	45.9 (0.51)	43.6 (0.53)	41.9 (0.54)	<0.0001
Height (cm)	169.4 (0.15)	165.6 (0.21)	167.9 (0.27)	170.4 (0.21)	173.8 (0.20)	<0.0001
Poverty-to-income ratio	3.1 (0.05)	2.9 (0.06)	3.2 (0.06)	3.2 (0.06)	3.1 (0.06)	<0.0001
Energy Intake (kcal/day)	2172.0 (12.9)	1431.9 (9.5)	1887.4 (12.7)	2276.1 (13.6)	3111.3 (20.1)	<0.0001
C-reactive protein (mg/dL)	0.36 (0.01)	0.38 (0.02)	0.35 (0.01)	0.33 (0.02)	0.36 (0.02)	0.30
Forced Expiratory Volume in 1 second (FEV_1_) (mL)	3251.2 (15.5)	2942.9 (22.5)	3116.2 (26.9)	3326.8 (19.6)	3604.5 (23.6)	<0.0001
Forced Vital Capacity (FVC) (mL)	4163.9 (14.9)	3754.8 (24.2)	3989.4 (29.8)	4258.4 (21.6)	4629.0 (23.6.)	<0.0001
FEV_1_/FVC ratio	0.78 (0.003)	0.78 (0.002)	0.78 (0.002)	0.78 (0.002)	0.78 (0.003)	0.56
FEV_1_% predicted	97.0 (0.41)	96.8 (0.53)	97.2 (0.56)	97.2 (0.50)	97.0 (0.50)	0.79
FVC% predicted	98.8 (0.31)	98.7 (0.44)	99.0 (0.56)	99.0 (0.56)	98.5 (0.49)	0.77
**Discrete Variables, *n* (%)**						
**Body Mass Index (BMI) Category (WHO classification)**						
<18.5	142 (1.3)	48 (1.7)	30 (1.2)	25 (0.77)	43 (1.6)	0.003
≤18.5–24.9	3105 (30.2)	948 (32.6)	763 (31.5)	753 (29.9)	685 (26.7)	
≥25–30	3675 (33.8)	1099 (32.7)	911 (34.2)	869 (32.8)	840 (35.2)	
<30 kg/m^2^	6898 (63)	1192 (32.9)	983 (32.9)	1048 (36.4)	893 (36.5)	
**BMI Category (obesity stratification)**						
≤30 kg/m^2^	6898 (63.0)	2091 (63.6)	1696 (67.0)	1640 (63.3)	1563 (63.1)	0.02
>30 kg/m^2^	4082 (37.0)	1196 (36.4)	991 (33.0)	1055 (39.7)	898 (36.8)	
≤40 kg/m^2^	10,388 (93.0)	3085 (94.6)	2517 (94.4)	2516 (93.4)	2277 (92.8)	0.07
>40 kg/m^2^	742 (7.0)	202 (5.4)	179 (5.6)	178 (6.6)	184 (7.2)	
**Race**						
Mexican American	1777 (16.1)	576 (8.9)	424 (7.5)	452 (7.5)	345 (7.6)	<0.001
Other Hispanic	1184 (10.6)	448 (6.9)	274 (4.9)	262 (4.9)	200 (4.1)	
Non-Hispanic White	5001 (44.7)	1176 (62.6)	1235 (70.7)	1265 (70.7)	1328 (76.1)	
Non-Hispanic Black	2306 (20.6)	706 (11.7)	561 (10.0)	561 (10.0)	471 (8.8)	
Other	892 (8.0)	399 (9.9)	206 (6.9)	161 (6.8)	126 (3.4)	
**Sex**						
Male	5548 (49.5)	1130 (31.4)	1150 (40.4)	1481 (53.0)	1787 (71.7)	<0.001
Female	5632 (50.5)	2175 (68.6)	1550 (59.6)	1224 (47.0)	683 (28.3)	
**Airflow obstruction**						
No	9756 (87.3)	2902 (86.8)	2351 (86.6)	2365 (87.0)	2138 (85.9)	0.78
Yes	1424 (12.7)	403 (13.2)	349 (13.4)	340 (13.0)	332 (14.1)	
GOLD 1 (FEV_1_/FVC < 0.7; FEV_1_ ≥ 80% predicted)	658 (53.9)	163 (52.2)	164 (56.2)	159 (51.6)	172 (58.2)	N/A
GOLD 2 (FEV_1_/FVC < 0.7; 50% ≤ FEV_1_ <80% predicted)	494 (40.0)	125 (39.4)	121 (38.6)	130 (43.6)	118 (38.3)	
GOLD 3 (FEV_1_/FVC < 0.7; 30% ≤ FEV_1_ 50% predicted)	67 (0.05)	25 (8.2)	15 (4.6)	18 (408)	9 (3.5)	
GOLD 4 FEV_1_/FVC < 0.7; FEV_1_ < 30% predicted)	2 (0.001)	1 (0.3)	1 (0.5)	0	0	
**Smoking Status**						<0.001
Never	5898 (54.5)	1940 (57.5)	1426 (56.3)	1380 (53.5)	1152 (51.6)	
Former	2491 (22.9)	693 (22.4)	643 (24.7)	631 (24.4)	524 (23.7)	
Current	2442 (22.5)	603 (20.2)	548 (19.0)	610 (22.1)	681 (24.7)	

**Table 2:** Results of multivariate regression models for saturated fat and lung function in adults with airflow obstruction (*n* = 1424).

Outcome	Quartile 1 <16.98 g/day		Quartile 2 >16.98 to 24.06 g/day		Quartile 3 >24.06 to 33.69 g/day		Quartile 4 >33.69 g/day
	β	*p*	β	*p*	β	*p*	
FEV_1_ *	−1264	0.04	−484	0.35	−931	0.14	Ref
FVC *	−16588	0.01	−624	0.44	−980	0.10	Ref
FEV_1_/FVC *	−0.005	0.16	−0.004	0.16	−0.001	0.58	Ref
FEV_1_ % predicted **	−2.8	0.15	−0.43	0.78	−2.20	0.20	Ref
FVC % predicted **	−3.3	0.04	−0.75	0.67	−1.7	0.17	Ref

* Adjusted for age, gender, race, height, total energy intake, family income to poverty ratio, BMI, smoking status, and CRP. ** Adjusted for total energy intake, family income to poverty ratio, BMI, smoking status, and CRP.

**Table 3:** (**a**) Ranges of intake of individual fatty acid by quartile for participants with airflow obstruction. (**b**) Results of multivariate regression analysis for the relationship between individual saturated fatty acids (SFA) intake and forced vital capacity (FVC) % predicted in individuals with chronic obstructive pulmonary disease (COPD).

**a**								
	**Entire population Mean (SE)**				**Quartile 1**	**Quartile 2**	**Quartile 3**	**Quartile 4**
Butanoic acid intake (C4:0), gm/day	0.66 (0.01)				<0.20	>0.20 to 0.40	>0.40 to 0.68	>0.68
Hexanoic acid intake (C6:0), gm/day	0.30 (0.005)				<0.12	>0.12 to 0.23	>0.23 to 0.39	>0.39
Octanoic acid intake (C8:0), gm/day	0.25 (0.004)				<0.10	>0.10 to 0.19	>0.19 to 0.33	>0.33
Decanoic acid intake (C10:0), gm/day	0.47 (0.007)				<0.20	>0.20 to 0.36	>0.36 to 0.58	>0.58
Dodecanoic acid intake (C12:0), gm/day	0.78 (0.02)				<0.26	>0.26 to 0.49	>0.49 to 0.87	>0.87
Tetradecanoic acid (C14:0), gm/day	2.3 (0.03)				<1.05	>1.05 to 1.73	>1.73 to 2.63	>2.63
Hexadecanoic acid (C16:0), gm/day	14.4 (0.14)				<8.01	>8.01 to 11.44	>11.44 to 15.90	>15.90
Octadecanoic acid (C16:0), gm/day	6.7 (0.07)				<3.54.01	>3.54 to 5.26	>5.26 to 7.46	>7.46
**b**								
FVC % Predicted *	**Quartile 1**		**Quartile 2**		**Quartile 3**		**Quartile 4**	***p_adj_* for linear trend**
	**β**	***p* value**	**β**	***p* value**	**β**	***p* value**		
Butanoic acid (C4:0)	−2.22	0.009	−1.53	.03	−1.26	0.04	Ref	0.01
Hexanoic acid (C6:0)	−1.61	0.04	−0.73	0.30	−0.83	0.13	Ref	0.05
Octanoic acid (C8:0)	−2.06	0.009	−1.53	0.02	−0.45	0.38	Ref	0.005
Decanoic acid (C10:0)	−1.86	0.03	−1.81	0.01	−1.12	0.04	Ref	0.03
Dodecanoic acid (C12:0)	−1.72	0.02	−1.07	0.07	−0.41	0.49	Ref	0.01
Tetradecanoic acid (C14:0)	−2.61	0.004	−2.05	0.01	−1.54	0.02	Ref	0.004
Hexadecanoic acid (C16:0)	−0.02	0.97	−0.17	0.79	−0.25	0.64	Ref	0.75
Octadecanoic acid (C18:0)	1.04	0.23	0.40	0.59	0.65	0.29	Ref	0.39

* Adjusted for total energy intake, family income to poverty ratio, BMI, smoking status, and CRP.
